# Gynecologists’ Knowledge of the Association Between Periodontal Health and Female Sex Hormones

**DOI:** 10.7759/cureus.4513

**Published:** 2019-04-20

**Authors:** Amal Al-Qahtani, Shahad M Altuwaijri, Huda Tulbah, Afnan Al-Fouzan, Amani Abu-Shaheen

**Affiliations:** 1 Prosthodontist, College of Dentistry, King Saud University, Riyadh, SAU; 2 Prosthodontics, College of Dentistry, King Saud University, Riyadh, SAU; 3 Miscellaneous, College of Dentistry, King Saud University, Riyadh, SAU; 4 Epidemiology and Public Health, King Fahad Medical City, Riyadh, SAU

**Keywords:** gynecologists, saudi arabia, female sex hormones, periodontal diseases

## Abstract

Objective: To assess gynecologists’ knowledge of the association between female sex hormones and periodontal health.

Methods: A cross-sectional study was conducted on gynecologists at various hospitals across the five areas of Riyadh, Saudi Arabia. Data was collected using a self-administered questionnaire. The questionnaire was divided into three parts, covering the participants’ demographics, their knowledge about the association between periodontal health and female sex hormones, and the participants’ practices regarding medications prescribed to treat their patients.

Results: During the study period, 203 gynecologists agreed to take part in this study. The overall mean percentage of knowledge regarding the association between periodontal health and female sex hormones among participants was 66.8%. Only 53.0% of participants reported that periodontal disease is a risk factor for preterm deliveries; 50.0% indicated that gingival changes could be induced by the long-term use of oral contraceptives and 35.1% agreed that periodontal health checkups must be regularly carried out for pregnant women.

Conclusions: Although the overall knowledge level of the gynecologists about periodontal health and female sex hormones was satisfactory, they showed an unsatisfactory level of knowledge about the association between periodontal disease and another aspect of women's health across their adult lifespan, such as the importance of a regular periodontal health checkup for pregnant women. Increased awareness amongst gynecologists may improve their level of knowledge about the effect and importance of periodontal health for women.

## Introduction

Periodontal diseases are a set of infectious diseases caused predominantly by different types of gram-negative anaerobic and microaerophilic bacteria [1]. Periodontal disease is one of the most common chronic infectious diseases: the overall prevalence ranges from 10% to 90% in adults [2-4], while among pregnant women, the prevalence varies from 10% to 74% [5-7]. Many factors may change the prevalence and severity of the disease [8]. Sex hormones are one of these factors, which likely affect the pathogenesis of periodontal diseases [9], as hormonal disturbance affects the physiology of host organism interactions in the oral cavity [10]. Among these, estrogen and progesterone have been most strongly linked to periodontal diseases [11, 12].

Progesterone and estrogen levels are higher during pregnancy compared to the menstrual cycle period [13]. During pregnancy, gingivitis is the most common oral manifestation [14]. Similar types of gingival changes are also seen in women who use oral contraceptives. Moreover, during menopause, when hormonal levels decline, women experience changes in the oral mucosa [10]. Several studies reported that periodontal disease affects pregnancy outcomes [15, 16]. A meta-analysis showed a significant risk of preterm delivery and low birth weight among pregnant women with periodontitis [17]. It has been reported that women with periodontitis have a five-times higher risk of preterm birth before 35 weeks of gestation and a seven-times higher risk of delivery before 32 weeks of gestation [18]. Similarly, in Saudi Arabia, a previous study reported a high prevalence of periodontal disease among pregnant women and a prevalence of preterm low birth weight babies of about 11% [19]. Moreover, a significant association between preeclampsia and periodontal disease has been reported in different studies [20,21]. Owing to the high prevalence of periodontal disease and its association with systemic disease, more attention must be considered from medical and public health sectors to improve the healthcare of women and children. Moreover, periodontal disease is preventable, and early intervention may decrease the microbial changes and improve periodontal health in hormonal-sensitive tissue. Furthermore, the integration of periodontal care with obstetric management may enhance pregnancy outcomes [22]. Gynecologists treat women across their adult life span and during these hormonal changes; therefore, it is essential that these doctors realize the effect of hormonal variations on a woman’s periodontal health and work to enhance the oral health of their patients. Currently, there is inadequate research involving the knowledge of gynecologists about the association between female sex hormones and periodontal health [19]. Thus, this study was conducted to assess the gynecologists’ knowledge of the association between female sex hormones and periodontal health.

## Materials and methods

Study design and setting

After obtaining approval (FR 0135) from the King Saud University, Riyadh, Research Ethics Committee, a cross-sectional study was conducted from December 2015 to December 2016 to assess gynecologists’ knowledge of the association between female sex hormones and periodontal health. The study was performed at various hospitals, clinics, and medical colleges across the five areas of Riyadh city, Saudi Arabia. The research ethics committee confirmed that informed consent from participants was not needed.

Participants

Gynecologists practicing at the time of the study at any of these hospitals, clinics, and medical colleges were eligible to participate in this study.

Recruitment

A simple random sampling technique was used to approach and invite gynecologists during the regular meetings over a six-month period to reach the needed sample size. Subjects were approached by the study-physicians to fill out the questionnaire.

Data collection tool

Data collection was accomplished using a self-administered questionnaire, which was developed based on an in-depth review of published studies that were conducted by Patil et al. and Rahman et al. [23, 24]. A pilot study was conducted on 25 participants to ensure the clarity of the questionnaire and to evaluate its reliability. Cronbach's alpha coefficient was used to evaluate the reliability of the questionnaire, which was < 0.70. Moreover, the validity of the questionnaire was ensured by a committee of experts in research methodology, obstetrics and gynecology, and periodontal health.

The questionnaire consisted of three parts. The first part gathered demographic data, including age, gender, and type of hospital (governmental, private, medical college). The second part explored the respondents’ knowledge regarding the following: (i) female sex hormones and their effect on periodontal health; (ii) whether microflora affect periodontal health or not; (iii) to whom should referral of any gingival tissue changes be addressed; (iv) effect of periodontal disease on preterm low birth weight deliveries; (v) effect of oral contraceptives on gingival health; (vi) whether treatment of gingival changes is needed; and (vii) the importance of periodontal health checkup for pregnant women. It included multiple choice questions, where each question was coded as correct=1 and incorrect=0. The third part explored the respondents’ practices regarding medications prescribed to treat their female patients with gingival enlargement or gingival bleeding.

Sample size calculation

Sample size calculation was based on the results of a pilot study that was conducted on 25 subjects to estimate the gynecologists' level of knowledge about the association between female sex hormones and periodontal health. The results showed that 75% of respondents had some knowledge regarding female sex hormones and periodontal health. This result permitted us to calculate the needed sample size of 203 subjects. The sample size was computed using the Raosoft online sample size calculator (Raosoft Inc., Seattle, USA) with a 5% margin of error and 90% confidence interval.

Statistical analysis

Data was analyzed using the IBM Statistical Package for the Social Sciences software (SPSS), Version 22.0 (IBM Corp. Armonk, NY, USA). A respondent's level of knowledge was calculated as a percentage of correct answers that were answered by the respondent. The data was analyzed using one-way analysis of variance (ANOVA) to compare the mean level of knowledge percent between the participants regarding their type of institution and age groups. While for gender, an independent t-test was used to compare the mean knowledge level percentage. Descriptive statistics was used to describe the medications prescribed by the study participants to treat their female patients with gingival enlargement or bleeding. A p-value <0.05 was considered statistically significant.

## Results

During the study period, 203 gynecologists agreed to take part in this study. One hundred twenty-three (60.6%) participants were from governmental hospitals while only 27 (13.3%) were from medical college hospitals. The overall mean knowledge percentage regarding the association between periodontal health and female sex hormones among participants was 66.8%. The statistical analysis of the participants’ demographics showed that age and gender had a significant association with participants’ mean knowledge percentage regarding periodontal health and female sex hormones (P < 0.000), (P <0.020), respectively (Table 1). Participants older than 50 years had greatest means of knowledge percentage regarding the association between periodontal health and female sex hormones compared to the other groups. The mean knowledge percentage was greater among male gynecologists (74.3±22.33) compared to female gynecologists (65.5±18.15).

**Table 1:** Participants’ mean knowledge percent regarding female sex hormones and periodontal health according to their demographic characteristics. * One-way analysis of variance (ANOVA); ** Independent t-test; Significant p-value is <0.05; CI: Confidence interval; SD: Standard deviation.

Variable	Number of participants (%)	Mean knowledge percent ± SD	95%CI		p-value
			Minimum	Maximum	
Age category (years)*					
30-40	75 (36.7%)	55.8±19.23	51.34	60.25	0.000
41-50	59 (29.1%)	67.8±22.76	61.87	73.73	
>50	69 (34.2%)	77.9±18.09	73.5	82.19	
Gender**					
Female	172 (84.7%)	65.5±18.15	62.17	68.89	0.020
Male	31 (15.3%)	74.3±22.33	67.51	81.06	
Type of hospital*					
Governmental	123 (60.6%)	64.7±21.59	60.84	68.55	0.200
Private	53 (26.1%)	70.1±22.14	63.89	76.22	
Medical college	27 (13.3%)	70.7±22.74	61.38	79.36	

The results presented in Table 2 revealed that 175 (86.2%) participants agreed that hormonal changes might induce gingival tissue changes across the female life cycle, while only 107 (53.0%) of the participants reported that periodontal disease is a risk factor for preterm low birth weight deliveries. Furthermore, 101 (50.0%) of the participants indicated that gingival changes were induced by the long-term use of oral contraceptive pills. Only 35.1% of the participants agreed that periodontal health checkups must be regularly carried out for pregnant women.

**Table 2:** Participants' knowledge of the association between periodontal health and female sex hormones.

	Number of agreed n (%)
Hormonal changes may induce gingival tissue changes across the female life cycle	175 (86.2)
Referrals and reports of gingival tissue changes to periodontist or dentist	180 (89.1)
Alteration in oral microflora was a primary cause of gingival tissue changes	164 (81.2)
Observed gingival changes need treatment	147 (72.8)
Periodontal disease is a risk factor for preterm low birth weight deliveries	107 (53.0)
Gingival changes induced by the long-term use of oral contraceptive pills	101 (50.0)
Periodontal health checkups must be regularly carried out for pregnant women	71 (35.1)

Table 3 presents the medications prescribed by the study participants to treat their female patients with gingival enlargement or bleeding. The majority (72.9%) of participants prescribed mouthwash for their patient, 37 (18.3%) prescribed vitamin supplements, and seven (3.5%) prescribed antibiotics. Only one (0.5%) participant reported not prescribing any medications to treat gingival enlargement or bleeding.

**Table 3:** Medications prescribed for patients with gingival enlargement or bleeding.

Treatment modalities	Number of participants (%)
Mouthwash	148 (72.9)
Vitamin supplements	37 (18.3)
Antibiotics	7(3.5)
Antiseptic gel	6 (3.0)
Analgesics	3 (1.5)
None	1 (0.5)

## Discussion

The present study exhibits valuable understanding concerning gynecologists’ knowledge of the association between periodontal health and female sex hormones. Although the participants showed an average overall level of knowledge about the association between periodontal health and female sex hormones, they showed an unsatisfactory level of knowledge about the association between periodontal diseases and preterm low birth weight deliveries. These results were similar to a study conducted by Shenoy et al. that reported that the respondents' knowledge regarding the oral manifestations of periodontal disease was high, whereas the perceptions of periodontal disease as a cause for preterm low birth weight deliveries was low [22]. Several studies have reported that subclinical infections amongst pregnant women are probably the most frequent cause of low birth-weight deliveries [25, 26]. This may be explained by periodontal disease, inflammatory mediators, and cytokine production in the maternal serum, which increases the risk for negative pregnancy outcomes. Periodontal infections may even target the placental membranes [15, 16]. Thus, periodontal disease not only influences women but also affects pregnancy outcomes.

The present study also showed that while 86.8% of the participants knew that hormonal changes might induce gingival tissue changes across the female life cycle, they were less aware of the importance of a regular periodontal health checkup for pregnant women. Likewise, a study conducted by AI Habashneh et al. in northern Jordan revealed that almost 68% of the respondents didn't recommend a periodontal evaluation for pregnant women as a part of antenatal care [27], although the majority of participants prescribed mouthwash, antibiotics, and vitamin supplements to their patients with gingival enlargement or bleeding.

Our results showed an unsatisfactory level of knowledge among the participants about the association between gingival changes induced by the long-term use of oral contraceptive pills. Thus, gynecologists should be educated more about the effect of long-term use of oral contraceptive pills on gingival tissue changes.

Our data indicated that male gynecologists demonstrated a greater level of knowledge of the association between periodontal health and female sex hormones compared to female gynecologists. Also, our study showed that older participants had the greatest overall level of knowledge compared to younger participants. This difference in the level of knowledge may be explained by the fact that older gynecologists have more experience and awareness regarding the association between periodontal health and female sex hormones.

Unfortunately, the concepts surrounding periodontal status and female sex hormones are not frequently addressed in routine practice. Thus, dental and medical school curricula should fundamentally stress on systemic medicine and better integrate information across health disciplines and clinical applications [28].

Gynecologists treat women; therefore, it is essential that these doctors realize the effect of hormonal variations on a woman’s periodontal health and work to enhance the oral health of their patients. Currently, there is inadequate research involving the knowledge of gynecologists about the association between female sex hormones and periodontal health [19]. Thus, this study was conducted to assess gynecologists’ knowledge of the association between female sex hormones and periodontal health.

An important strength of this study was that our sample included participants from various governmental and private hospitals, clinics and medical colleges across the five areas of Riyadh city, Saudi Arabia.

Our results can be a guide to policymakers to include screening and prevention of periodontal disease among women in maternal and child health care programs, which can lead to the avoidance of the possible adverse effects of this disease on women's oral health and pregnancy outcomes.

## Conclusions

In conclusion, the current study assesses the level of knowledge of the association between periodontal health and female sex hormones among gynecologists. Although the overall knowledge level of gynecologists about periodontal health and female sex hormones was satisfactory, they showed an unsatisfactory level of knowledge about the association between periodontal disease and another aspect of women's health across their adult lifespan, such as the importance of a regular periodontal health checkup for pregnant women. Increased awareness amongst gynecologists may improve their level of knowledge about the effect and importance of periodontal health for women.
